# Supplementary material for: Bacterial OTU deubiquitinases regulate substrate ubiquitination upon Legionella infection
Source: eLife. 2020 Nov 13;9:e58277. doi: 10.7554/eLife.58277 (PMC7690952; doi:10.7554/eLife.58277)
Supplement: Supplementary file 1. [file elife-58277-supp1.docx]

**Data collection and refinement statistics**

|  | **LotC_14-310_** |
| --- | --- |
| **Data Collection** |  |
| Wavelength (Å) | 1.070333 |
| Space group | P 1 21 1 |
| Cell dimensions |  |
| a, b, c (Å) | 38.534 140.979 57.437 |
| 𝛼, 𝛽, 𝛾 (°) | 90, 90, 90 |
| *R*_merge_ | 0.03065 (0.3765) |
| *R*_pim_ | 0.03065 (0.3765) |
| CC_1/2_ | 0.999 (0.811) |
| CC^*^ | 1 (0.946) |
| I / 𝜎I | 14.85 (1.96) |
| Completeness | 99.23 (99.52) |
| Redundancy | 2.0 (2.0) |
| **Refinement** |  |
| Resolution (Å) | 44.53 – 2.42 (2.506 -2.42) |
| No. reflections | 46248 (4550) |
| *R*_work_/*R*_free_ | 0.2285 /0.2852 |
| No. atoms | 4391 |
| Macromolecules | 4380 |
| ligands | 10 |
| Solvent | 1 |
| B-factors | 76.55 |
| Macromolecules | 76.14 |
| ligands | 256.24 |
| Solvent | 79.91 |
| R.m.s deviations |  |
| Bond lengths (Å) | 0.008 |
| Bond angles (°) | 1.00 |
